# A Small Cysteine-Free Protein Acts as a Novel Regulator of Fungal Insect-Pathogenic Lifecycle and Genomic Expression

**DOI:** 10.1128/mSystems.00098-21

**Published:** 2021-03-23

**Authors:** Ya-Ni Mou, Bo Fu, Kang Ren, Sheng-Hua Ying, Ming-Guang Feng

**Affiliations:** a MOE Laboratory of Biosystems Homeostasis and Protection, College of Life Sciences, Zhejiang University, Hangzhou, Zhejiang, China; Oak Ridge National Laboratory

**Keywords:** entomopathogenic fungi, gene expression and regulation, putative secreted proteins, virulence determinant

## Abstract

Small secreted proteins (SSPs), particularly cysteine-rich proteins secreted during fungal infection, comprise virulence effectors in plant-pathogenic fungi but remain unknown in insect-pathogenic fungi. We report here that only a small cysteine-free protein (CFP) is indispensable for insect pathogenicity of Beauveria bassiana among 10 studied SSPs (99 to 274 amino acids [aa]), including seven hypothetical proteins containing 0 to 12 Cys residues. CFP (120 aa) features an N-terminal signal peptide (residues 1 to 17), a nuclear localization signal motif (residues 24 to 57), and no predictable domain. Its homologs exist exclusively in insect-pathogenic Cordycipitaceae and Clavicipitaceae. Fluorescence-tagged CFP fusion protein was localized in the nucleus but extracellularly undetectable, suggesting an inability for CFP to be secreted out. Disruption of *cfp* resulted in abolished pathogenicity via normal cuticle infection, attenuated virulence via hemocoel injection, compromised conidiation capacity versus little growth defect, impaired conidial coat, blocked secretion of cuticle-degrading enzymes, impeded proliferation *in vivo*, disturbed cell cycle, reduced stress tolerance, and 1,818 dysregulated genes (genomic 17.54%). Hundreds of those genes correlated with phenotypic changes observed in the disruption mutant. Intriguingly, nearly 40% of those dysregulated genes encode hypothetical or unknown proteins, and another 13% encode transcription factors and enzymes or proteins collectively involved in genome-wide gene regulation. However, purified CFP showed no DNA-binding activity in an electrophoretic mobility shift assay. These findings unveil that CFP is a novel regulator of fungal insect-pathogenic life cycle and genomic expression and that cysteine richness is dispensable for distinguishing virulence effectors from putative SSPs in B. bassiana.

**IMPORTANCE** Small cysteine-rich proteins secreted during plant-pathogenic fungal infection comprise virulence effectors. Our study confirms that only a cysteine-free protein (CFP) is determinant to insect-pathogenic fungal virulence among 10 small putatively secreted proteins containing 0 to 12 Cys residues. Disruption of *cfp* abolished insect pathogenicity and caused not only a series of compromised cellular events associated with host infection and disease development but also dysregulation of 1,818 genes, although no DNA-binding activity was detected in purified CFP samples. Nearly 13% of those genes encode transcription factors and enzymes or proteins collectively involved in transcriptional regulation. Altogether, CFP serves as a novel regulator of the fungal insect-pathogenic life cycle and genomic expression. Cysteine richness is dispensable for distinguishing virulence effectors from the fungal SSPs.

## INTRODUCTION

Filamentous fungal insect pathogens serve as main sources of fungal insecticides and acaricides that are environment friendly (1, 2) and safe to apiculture (3). Approximately 15% genes in the fungal genomes encode putative virulence determinants (4–7), which are presumably involved in fungus-insect interactions crucial for fungal infection and lethal action. However, only a few of those have been characterized.

Fungus-insect interactions start from conidial adhesion to insect cuticle, which is impregnated with chitin and proteins and covered with an epicuticular layer composed of lipids and hydrocarbons (5, 8, 9). Conidial germination leads to formation of germ tubes and invasion of hyphae into the host body through cuticular penetration by secreted cuticle-degrading enzymes, such as subtilisin-like Pr1 family proteases (10), and/or mechanic pressure of appressoria formed at hyphal tips. Such appressoria are often seen in Metarhizium spp. (11, 12) but rarely in typical Beauveria bassiana strains, implicating somewhat distinctive mechanisms underlying the host infection of the *Beauveria* lineage that could have evolved insect pathogenicity from plant pathogens or endophytes approximately 130 million years earlier than the Metarhizium lineage (7). The fungal cuticle infection is initiated by G protein-coupled receptors, which mediate host recognition and the activation of downstream pathways to regulate appressorial formation in Metarhizium spp. (4, 12) or developmental and genetic networks in B. bassiana (13), and ultimately leads to hyphal entry into host hemocoel. The mentioned receptors constitute a large family of transmembrane receptors responsible for transduction of external signals into intracellular responses in association with complicated signaling networks (14). Upon entry into hemocoel, hyphae encounter, and hence must overcome, cellular and humoral immune responses of the host (9, 15) while they turn into unicellular thin-wall hyphal bodies (blastospores) to facilitate intrahemocoel proliferation by yeast-like budding until host death from mummification by fungal cells (16–18). In the dying host, hyphal bodies turn back into hyphae to penetrate the cuticle again for outgrowth on insect cadaver surfaces, where conidia are produced for a new infection cycle or dispersal/survival in host habitats. For an insect-pathogenic fungus, therefore, its ability to grow out of insect cadavers for conidiation can be considered an excellent indicator of its enabling host infection via cuticular penetration. In B. bassiana, dimorphic (hypha-blastospore and vice versa) transition critical for fungal kill action and infection cycle is an asexual development process controlled by the key activator *brlA* or *abaA* of the central developmental pathway (CDP) (19) and also orchestrated by other factors involved in transcriptional activation of *brlA* or *abaA* (20–24). The factors required for or associated with asexual development comprise extraordinary virulence factors, because single-gene disruptions may result in abolished or nearly abolished hyphal invasion into host body via cuticular penetration and attenuated virulence via hemocoel injection for cuticle-bypassing infection.

Small secreted proteins (SSPs), particularly cysteine-rich proteins secreted during fungal infection, are a class of presumable key virulence effectors in insect mycopathogens (7, 9) but have not been explored as well as those involved in pathogen-plant interactions (25–27). In plant-pathogenic fungi, small cysteine-rich proteins have been characterized as important virulence effectors, such as AvrII, Avr4, Avr4E, and Avr9 to be recognized by resistant proteins in the host plant (28, 29) and Ecp1, Ecp2, Ecp4, and Ecp5e as extracellular proteins to invoke a hypersensitive response of host plant to arrest fungal growth (30, 31). Revealed in a previous transcriptomic analysis, several hundreds of genes encoding secreted and nonsecreted proteins were induced in the first 48-h infection course of B. bassiana penetrating the cuticle of Plutella xylostella and colonizing the pest hemocoel (32), implicating their involvements in the fungus-insect interaction. Some of those genes have proved determinant to virulence due to reduced or even abolished pathogenicity in the absence of each. The characterized genes encode the vacuolar protein VLP4 (33), cyclophilin B (CypB) (34), and three nonsecreted proteins, including the class II lysyl-tRNA synthetase KRS (35), cytoplasmic Rei1 as a pre-60S subunit export factor (36), and nuclear Ssr4 as a cosubunit of chromatin-remodeling SWI/SNF and RSC complexes (37). Among those, only VLP4 is a small cysteine-rich protein (13 Cys resides in 230 amino acids [aa]). Many more identified SSPs remain unexplored. This study seeks to identify possible virulence effectors from 10 selected SSPs (99 to 274 aa), which share a predicted N-terminal signal peptide (NSP) and are presumably secreted in the fungal infection course. Our goal is to test the hypothesis by clarifying whether, how, and why SSPs are determinant to the fungal virulence denoted by median lethal actions of single-gene disruption mutants against a model insect. Among the 10 SSPs characterized in this study, only a small cysteine-free protein (CFP; previously named PspA-putative secreted protein A) proved determinant to virulence of B. bassiana. As presented below, CFP is involved in a wide array of cellular events and required for the fungal infection cycle and genome stability.

## RESULTS

### Recognition and sequence features of fungal CFP homologs.

**FIG 1:**
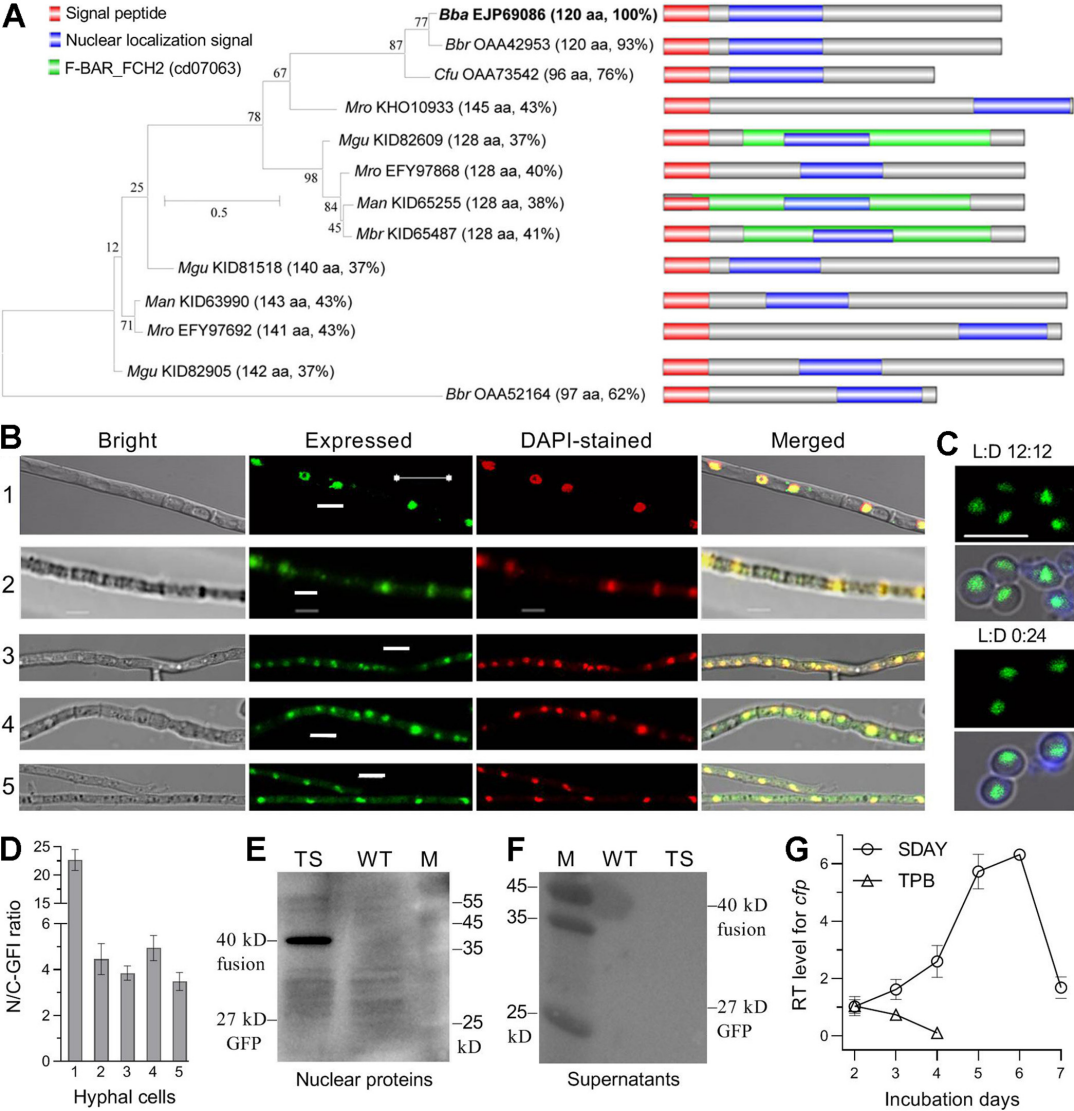
Sequence feature, subcellular localization, and transcriptional profile of CFP in B. bassiana. (A) Sequence features and phylogenetic ties of CFP homologs found in insect-pathogenic Cordycipitaceae (*Bba*, B. bassiana; *Bbr*, B. brongniartii; *Cfu*, Cordyceps fumosorosea) and Clavicipitaceae (*Man*, Metarhizium anisopliae; *Mbr*, Metarhizium brunneum; *Mgu*, Metarhizium guizhouense; *Mro*, Metarhizium robertsii). Each abbreviated fungal name is followed by the NCBI accession code of a CFP homolog, the length of its amino acid sequence, and its sequence identify to that of *Bba* CFP. Scale, branch length proportional to genetic distance assessed with the maximum likelihood method in MEGA7 at http://www.megasoftware.net/. (B and C) LSCM images (bar, 5 μm) for subcellular localization of GFP-tagged CFP fusion protein in hyphae and conidia (produced at L:D 12 h:12 h [light] or in the dark). Rows 1 to 5 are the hyphae stained with DAPI after being collected from a normal SDBY culture (control), the culture triggered for 12 h in CDB free of carbon and nitrogen sources, the cultures triggered for 90 min with 4 mM H_2_O_2_ and 0.4 M NaCl in one-fourth SDBY, and the culture in PBS irradiated at the UVB dose of 1 J/cm^2^, respectively. (D) Nuclear versus cytoplasmic green fluorescence intensity (N/C-GFI) ratios of the fusion protein in the hyphae of rows 1 to 5. (E and F) Western blots for signal of the fusion protein detected in the nuclear protein extracts from the 60-h-old SDBY cultures of the WT and transgenic strain (TS) and secreted into the supernatants of the cultures. (G) Relative transcript (RT) levels of *cfp* in the WT cultures during 7-day incubation on SDAY or 4-day incubation in TPB with respect to a standard at the end of 2-day incubation. Error bars, standard deviations (SD) of the means from 10 hyphae (D) or three cDNA samples (F).

The CFP sequence (NCBI accession EJP69086) annotated as hypothetical protein in the B. bassiana genome (5) consists of 120 aa (13.18 kDa), features an NSP (residues 1 to 17) and a nuclear localization signal (NLS) motif (residues 24 to 57) adjacent to the NSP but contains no Cys residue. The CFP sequence also does not contain any known function domain predictable at https://www.ncbi.nlm.nih.gov/Structure/ or http://smart.embl-heidelberg.de/. BLASTp analysis with the query CFP sequence resulted in recognition of one or two homologs (96 to 120 aa) in Beauveria brongniartii and Cordyceps fumosorosea (Cordycipitaceae) and one to three homologs (128 to 145 aa) in four Metarhizium species (Clavicipitaceae) (Fig. 1A). These insect pathogens usually undergo asexual cycles. Unexpectedly, no homolog was found in other entomopathogenic (Ascosphaera apis, Cordyceps militaris, *Cordyceps confragosa*, Moelleriella libera, and Sporothrix insectorum) and nonentomopathogenic (*Aspergillus*, *Botrytis*, *Fusarium*, *Neurospora*, and *Pyricularia*) ascomycetes. The identified CFP homologs (see Fig. S1A in the supplemental material) fall into distinct phylogenetic clades and share a similar NSP (17 aa) and an NLS (29 to 35 aa), and most of them lack any predictable domain. Exceptionally, three of nine Metarhizium homologs share a unique F-BAR_FCHO2 domain. The F-BAR (FES-CIP4 homology and bin/amphiphysin/Rvs) domain exists in proteins involved in membrane dynamics and actin reorganization, while the FCHO2 (FCH domain only 2 protein) domain remains functionally unknown (38). WoLF PSORT analysis (https://wolfpsort.hgc.jp/) revealed a possible involvement of B. bassiana CFP in extracellular activity and membranes and/or lumens of mitochondria, Golgi apparatus, peroxisomes, and endoplasmic reticulum (Fig. S1B) but failed to clue a link of CFP to the nucleus as predicted at http://nls-mapper.iab.keio.ac.jp/.

10.1128/mSystems.00098-21.1 FIG S1 Amino acid sequences of fungal CFP homologs and predicted cellular activities of CFP in B. bassiana. (A) Sequence alignment analysis of insect-pathogenic fungal CFP homologs with DNAman 8.0 at http://www.bio-soft.net/format/DNAMAN.htm/. (B) Extracellular and intracellular activities of B. bassiana CFP (EJP69086) predicted with WoLF PSORT (https://wolfpsort.hgc.jp/). Download FIG S1, JPG file, 1.9 MB. Copyright © 2021 Mou et al. 2021 Mou et al. https://creativecommons.org/licenses/by/4.0/ This content is distributed under the terms of the Creative Commons Attribution 4.0 International license.

These analyses implicate that CFP is distinct from those cysteine-rich virulence effectors in plant-pathogenic fungi (25, 27) and that its homologs seem to exist only in some asexual insect pathogens, particularly the *Beauveria* and Metarhizium lineages, as main sources of fungal pesticides (1).

### Subcellular localization and transcriptional profile of CFP in B. bassiana.

The predicted NLS implicates a nuclear localization of CFP. This implication was clarified by subcellular localization of a green fluorescence protein (GFP)-tagged CFP fusion protein (CFP::GFP) expressed in the wild-type strain B. bassiana ARSEF 2860 (designated WT). Revealed by laser scanning confocal microscopy (LSCM), CFP::GFP localized exclusively in the nuclei of hyphal cells collected from a 60-h-old Sabouraud dextrose broth plus yeast extract (SDBY) culture and stained with the nucleus-specific dye 4′,6-diamidino-2-phenylindole (DAPI) (row 1 in Fig. 1B) and also in the nuclei of aerial conidia produced on SDBY plus agar (SDAY) at an optimal regime of 25°C in a light/dark (L:D) cycle of 12 h:12 h or in full darkness (Fig. 1C). For subcellular responses of CFP to stress cues, hyphae from 48-h-old SDBY cultures were incubated for 12 h in Czapek-Dox broth (CDB) deficient of carbon and nitrogen sources, and those from 58-h-old cultures were triggered for 90 min with H_2_O_2_ (4 mM) or NaCl (0.4 M) in one-fourth SDBY (one-fourth nutrition strength of SDBY) or exposed to UVB irradiation (1 J/cm^2^) after being suspended in phosphate-buffered saline (PBS). As a result, CFP::GFP accumulated heavily in the nuclei and weakly in the cytoplasm comprising organelles (rows 2 to 5 in Fig. 1B). The mean ratio (22.7) of nuclear versus cytoplasmic green fluorescence intensities (N/C-GFI) quantified from the hyphae not triggered with any stress cue was 4.6- to 6.5-fold of those from the hyphae triggered with the stress cues of carbon/nitrogen starvation, high osmolarity, oxidation, and UVB irradiation (Fig. 1D). Western blotting with an anti-GFP antibody confirmed the presence of CFP::GFP in the nuclear protein extract isolated from the 60-h-old SDBY culture (Fig. 1E) but revealed no signal for its presence in the precipitant of the culture supernatant (Fig. 1F), suggesting a nonsecreted status of CFP. Moreover, transcription of *cfp* was increasingly upregulated in the WT culture during 7-day incubation on SDAY at the optimal regime and peaked on day 6 (Fig. 1G). In contrast, its transcript level in the WT cultures grown in trehalose-peptone broth (TPB) mimicking insect hemolymph (18, 33) peaked on day 2 and declined to a minimum on day 4, suggesting a status of its induced expression during early colonization of host hemocoel.

These data indicate that CFP is a nucleus-specific protein that may partially migrate to the cytoplasm in response to stress cues. The stress-responsive migration is likely associated with the activities of those organelles predicted with WoLF PSORT. Taken together with transcript changes, a nuclear localization of CFP under normal and stressful conditions implicates its involvement in the fungal nuclear events.

### CFP is indispensable for fungal pathogenicity and virulence.

**FIG 2:**
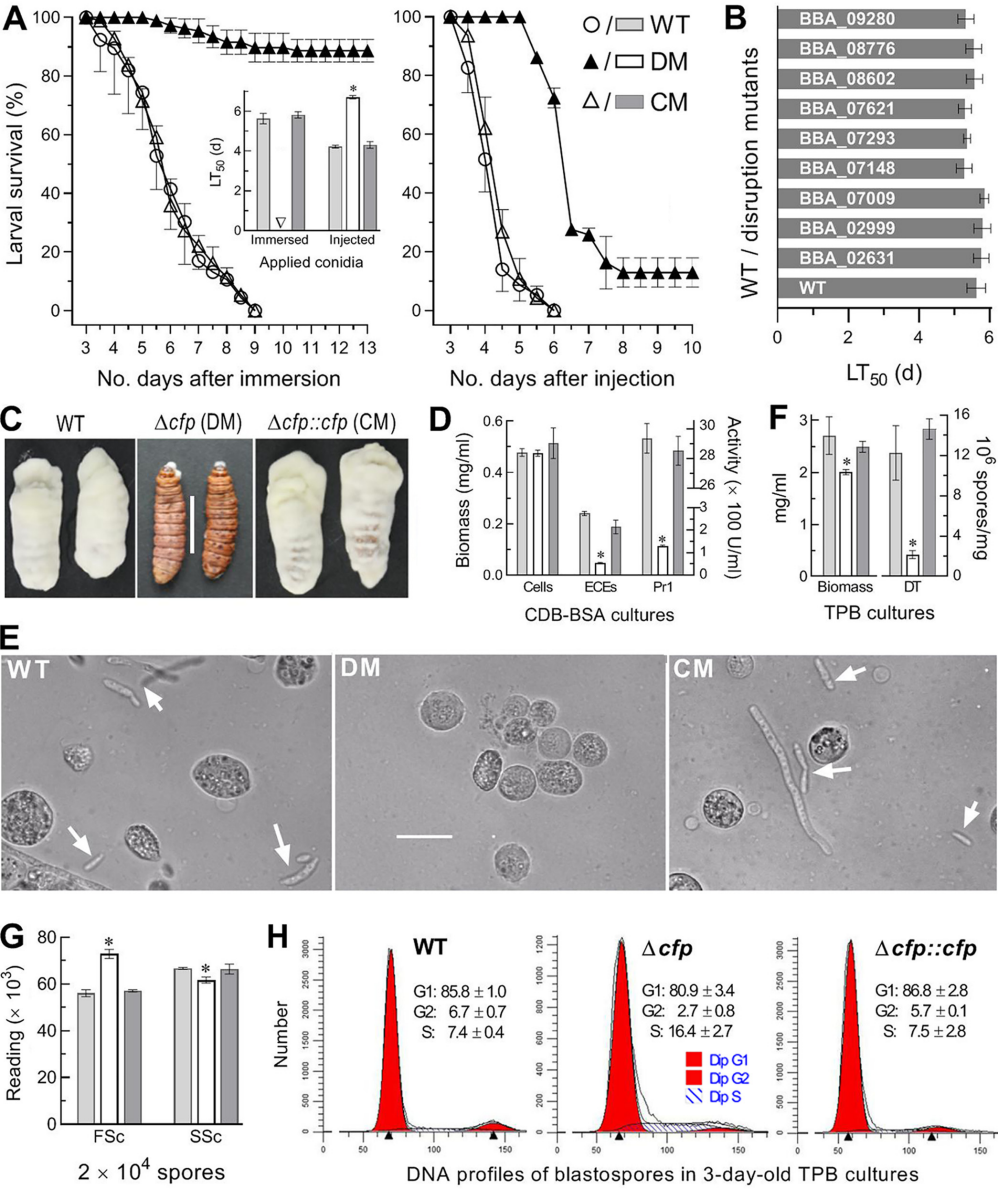
Indispensability of CFP for insect pathogenicity and virulence-related cellular events of B. bassiana. (A) Time-survival trends and LT_50_ estimates (no. of days) of G. mellonella larvae after topical application (immersion) of a 10^7^-conidia/ml suspension for NCI and intrahemocoel injection of ∼500 conidia per larva for CBI. (B) LT_50_ estimates for disruption mutants of nine SSP genes against the larvae via NCI. (C) Images (bar, 1 cm) for fungal outgrowths on the surfaces of insect cadavers 6 days after death from injection. (D) Biomass levels and total ECEs and Pr1 activities (U/ml) assessed from 3-day-old CDB-BSA cultures and their supernatants. (E) Microscopic images (bar, 20 μm) for the presence of hyphal bodies (arrows) and host hemocytes (spherical or subspherical cells) in hemolymph samples taken from surviving larvae 6 days post-NCI. (F) Biomass levels and dimorphic transition (DT) rates (no. blastospores/mg biomass) measured from the 3-day-old cultures of a 10^6^-conidia/ml suspension in TPB mimicking insect hemolymph. (G) Mean size and complexity of 2 × 10^4^ blastospores indicated by the FSc and SSc readings via flow cytometry. (H) FACS analysis of 2 × 10^4^ DNA-stained blastospores for distribution of their DNA profiles indicating cell cycle phases (mean ± SD). *, *P* < 0.05 in Tukey’s HSD test. Error bars, SDs of the means from three independent replicates.

The disruption and complementation mutants of *cfp* were created as described in Materials and Methods and identified via PCR and Southern blot analyses with paired primers and amplified probe (see Fig. S2). The Δ*cfp* mutant lost almost all ability to infect Galleria mellonella fourth-instar larvae via normal cuticle infection (NCI) initiated by topical application (immersion) of a 10^7^-conidia/ml suspension and also was compromised severely in virulence via cuticle-bypassing infection (CBI) by intrahemocoel injection of ∼500 conidia per larva, as indicated by time-survival trends in either infection mode and median lethal time (LT_50_) estimated from the resultant time-mortality records (Fig. 2A). The control (WT and complemented) strains caused 100% mortality within 9 days and a mean LT_50_ of 5.72 days via NCI, contrasting to only ∼10% mortality caused by Δ*cfp* 13 days post-NCI. The Δ*cfp* LT_50_ via CBI was prolonged by 57% versus a mean of 4.26 days for the control strains. In contrast, single-gene disruptions of nine other SSPs resulted in insignificant LT_50_ changes (Tukey’s honestly significant difference [HSD], *P* > 0.05) via NCI (Fig. 2B). These SSPs included hydrophobin-like protein (BBA_02999), alkaline foam protein B precursor (BBA_07009), LysM domain-containing protein (BBA_08602), and six hypothetical proteins containing 0 (BBA_09280), 1 (BBA_07293), 4 (BBA_07148, BBA_07621, and BBA_08776) and 12 (BBA_02631) Cys residues. Due to unaffected virulence, the nine mutants were not studied further. An emphasis turned to explore why *cfp* is required for fungal insect pathogenicity and virulence-related cellular events postinfection.

10.1128/mSystems.00098-21.2 FIG S2 Generation and identification of *cfp* mutants in B. bassiana. (A) Schematic diagram for the disruption strategy of *cfp*. (B) Paired primers used for manipulation of *cfp*. Underlined regions denote introduced cleavage sites of restriction enzymes for construction of *cfp*::*gfp* fusion (XmaI/HpaI) or homologous recombination of 5' and 3' *cfp* fragments separated by *bar* (EcoRI/BamHI and XbaI/HpaI) for targeted gene disruption. Italicized and underlined regions are the fragments for gateway exchange at the sites of HindIII/XbaI (in bold) for targeted gene complementation. (C) *cfp* mutants identified through PCR (lanes 1 to 3; M, DNA ladder) and Southern blot (lanes 4 to 6) analyses with paired primers and amplified probe (477 bp). Lanes 1 and 4, parental WT; lanes 2 and 5, Δ*cfp* mutant.; lanes 3 and 6, Δ*cfp*::*cfp* mutant. The detected PCR bands denote the fragments of 1,922 bp in the Δ*cfp* mutant, 1,263 bp in WT, and both of them in the Δ*cfp*::*cfp* strain, indicating that *cfp* was disrupted by the deletion of its partial coding sequence (189 bp) and promoter region (112 bp). Genomic DNA for Southern blotting of *cfp* was digested with SpeI/SpeI at the arrowed sites in the diagram. Download FIG S2, JPG file, 0.7 MB. Copyright © 2021 Mou et al. 2021 Mou et al. https://creativecommons.org/licenses/by/4.0/ This content is distributed under the terms of the Creative Commons Attribution 4.0 International license.

The role of *cfp* in NCI was revealed by fungal outgrowths on the CBI-killed larvae. The control strains grew readily out of insect cadavers and formed a heavy layer of hyphal mass on their surfaces 8 days postdeath, contrasting to an inability for the Δ*cfp* mutant to grow out (Fig. 2C). The inability implicated an essentiality of *cfp* for either intrahemocoel hyphae to penetrate through insect cuticle for outgrowth or hyphal invasion into insect body via NCI. Next, total activities of extracellular (proteolytic, chitinolytic, and lipolytic) enzymes (ECEs) and Pr1 family proteases collectively required for cuticle degradation during host infection (10) were quantified from the supernatants of submerged cultures from 3-day shaking incubation of a 10^6^-conidia/ml suspension in CDB containing bovine serum albumin (BSA) as the sole nitrogen source and enzyme inducer. Despite little change in biomass level, total ECEs and Pr1 activities decreased by 80% and 96%, respectively, in the Δ*cfp* mutant versus that in WT cultures (Fig. 2D), suggesting a main cause for the mutant inability to infect insects and grow out of cadavers through cuticular penetration.

To observe the proliferation of hyphal bodies in host hemocoel, hemolymph samples taken from surviving larvae 6 days post-NCI were examined under a microscope. Budding hyphal bodies were present in the samples of the larvae infected by two control strains but not in those of the mutant-infected larvae (Fig. 2E). This observation implicated impeded development of hyphal bodies *in vivo*, which was supported by reduced blastospore production in the 3-day-old cultures of a 10^6^-conidia/ml TPB mimicking insect hemolymph. Compared to the WT cultures, the Δ*cfp* cultures showed 87% reduction in blastospore production but only 26% biomass decrease (Fig. 2F), indicating a 61% contribution of *cfp* to dimorphic transition *in vitro*. The latter estimate coincided well with a lethal action of the mutant prolonged by 57% through CBI. Revealed by flow cytometry, the mutant blastospores displayed altered size and complexity (Fig. 2G) and disturbed cell cycle with shortened G_1_ and G_2_ phases and a prolonged S phase (Fig. 2H).

Altogether, CFP is indispensable for insect pathogenicity and virulence of B. bassiana. The indispensability relies upon its essential role in secretion of NCI-required enzymes and also in dimorphic transition to accelerate proliferation *in vivo* and host death.

### CFP is dispensable for hyphal growth but functional in the stress response.

**FIG 3:**
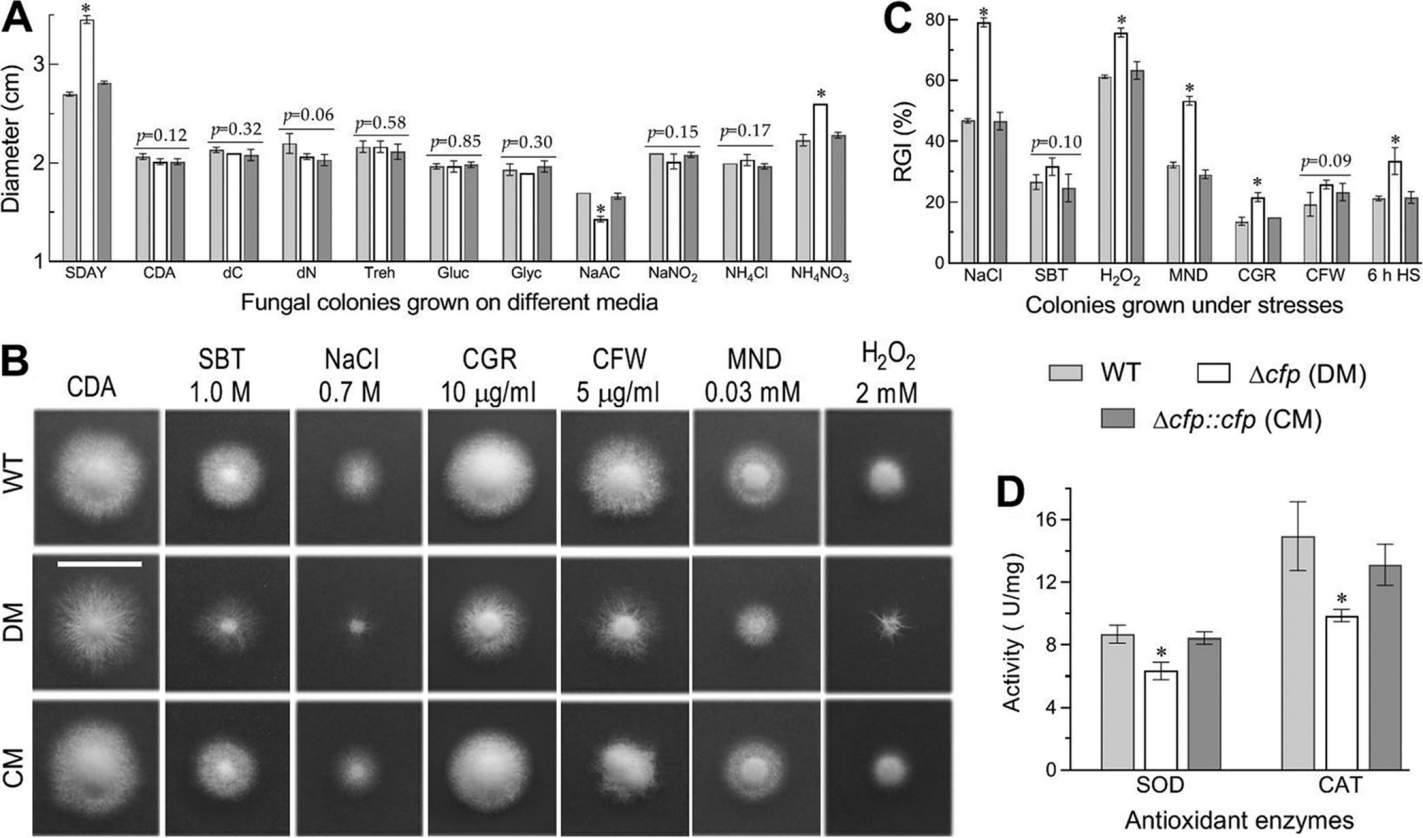
Impacts of *cfp* disruption on radial growth and stress response of B. bassiana. (A) Diameters of fungal colonies grown at 25°C for 8 days on rich SDAY, minimal CDA, and CDAs amended with different carbon (trehalose, glucose, glycerol, or sodium acetate [NaAc]) or nitrogen sources and deleted carbon source (dC) or nitrogen source (dN). (B and C) Images (scale, 2 cm) and relative growth inhibition (RGI) percentages of fungal colonies grown at 25°C for 8 days on CDA containing indicated concentrations of chemical stressors (SBT, sorbitol; CGR, Congo red; CFW, calcofluor white; MND, menadione). All colonies were initiated by spotting 1-μl aliquots of a 10^6^-conidia/ml suspension. (D) Total SOD and catalase (CAT) activities quantified in the protein extracts of 3-day-old SDAY cultures. *, *P* < 0.05 in Tukey’s HSD test. Error bars, SDs of the means from three independent replicates.

To explore the possible effects of *cfp* disruption on hyphal growth and invasion into host body, fungal colonies were initiated by spotting 1-μl aliquots of a 10^6^-conidia/ml suspension on the plates of rich SDAY, minimal CDB plus agar (CDA) and CDAs amended with different carbon or nitrogen sources. After an 8-day incubation at the optimal regime, little variability in colony size was found among the tested strains on the tested media (Fig. 3A). Exceptionally, the Δ*cfp* mutant growth was facilitated significantly on SDAY and the nitrogen source NH_4_NO_3_ and moderately suppressed only on sodium acetate as the carbon source. These data indicated that *cfp* was dispensable for hyphal growth on the scant media like oligotrophic insect integument.

The fungal kill action relies upon an ability to overcome stresses generated from host immune responses during cuticular penetration and subsequent hemocoel colonization (19, 39). Thus, fungal colonies were initiated as aforementioned on CDA containing chemical stressors. Compared to the control strains, the Δ*cfp* mutant became more sensitive to NaCl, menadione, H_2_O_2_, and Congo red during hyphal growth but showed null response to the noncation osmotic agent sorbitol or the cell wall antagonist calcofluor white (Fig. 3B and C). The mutant also showed increased sensitivity to 6-h heat shock at 42°C during the 6-day growth recovery at 25°C after exposure of 2-day-old SDAY colonies to 42°C. Its increased sensitivity to oxidative stress coincided well with 27% and 34% reductions in total activities of superoxide dismutases (SODs) and catalases (Fig. 3D), respectively, required for removal of superoxide anions and H_2_O_2_ (19).

### Importance of CFP for conidiation capacity and conidial quality.

**FIG 4:**
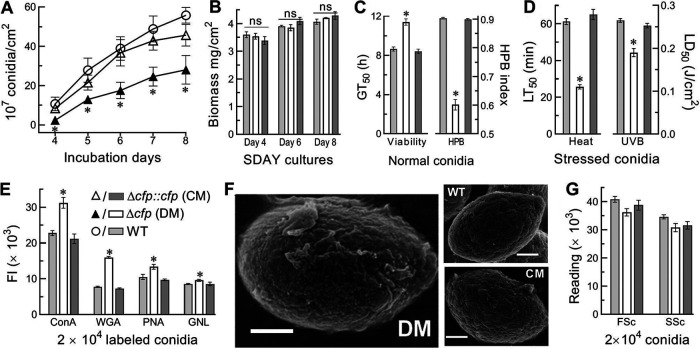
Impacts of *cfp* disruption on conidiation capacity and conidial quality of B. bassiana. (A and B) Conidial yields and biomass levels measured from the SDAY cultures during an 8-day incubation at the optimal regime of 25°C and L:D 12 h:12 h. The cultures were initiated by spreading 100 μl of a 10^7^-conidia/ml suspension per plate. (C) GT_50_ for 50% of conidial germination at 25°C and conidial hydrophobicity (HBP) index assessed in an aqueous-organic system. (D) LT_50_ and LD_50_ for conidial tolerance to 45°C wet-heat stress and UVB irradiation, respectively. (E) Fluorescence intensities (FI) for changes of carbohydrate epitopes on the surfaces of conidia labeled with Alexa Fluor 488-labeled lectins. (F) SEM images (bars, 0.5 μm) for conidial coat, which is well defined with an outermost layer of hydrophobin rodlet bundles in WT or CM but impaired severely in DM. (G) Mean size and complexity of conidia indicated by the FSc and SSc readings via flow cytometry. *, *P* < 0.05. Error bars, SDs of the means from three independent replicates.

Conidiation capacity and conidial quality are important for large-scale production of fungal pesticides. The conidial yield measured from the SDAY cultures initiated by spreading 100 μl of a 10^7^-conidia/ml suspension per plate at the optimal regime decreased by 77% and ∼50% in the Δ*cfp* mutant versus that in the WT on days 4 and 5 to 8, respectively (Fig. 4A), despite little difference in their biomass levels during the 8-day incubation (Fig. 4B). Moreover, several indices of conidial quality were compromised in the Δ*cfp* mutant relative to that in the WT, including a 33% decrease in hydrophobicity and 32% increase in median germination time (GT_50_) at 25°C (Fig. 4C) and 58% and 23% reductions in median lethal time (LT_50_) and dose (LD_50_) for tolerance to 45°C wet-heat stress and UVB irradiation, respectively (Fig. 4D). Furthermore, carbohydrate epitopes of conidial surfaces with pathogen-associated molecule patterns (PAMPs) to be perceived by host PAMP-recognition receptors (9, 25) were probed in fluorescent lectin-binding assays. The fluorescence intensity changes of labeled Δ*cfp* versus WT conidia demonstrated 37%, 106%, 27%, and 12% increases in the contents of α-glucose and α-*N*-acetylglucosamine (GlcNAc), β-GlcNAc and sialic acid residues, β-galactose, and mannose residues labeled by concanavalin A (ConA), wheat germ agglutinin (WGA), peanut agglutinin (PNA), and Galanthus nivalis lectin (GNL), respectively (Fig. 4E). Previously, the conidial coat of B. bassiana was characterized as a well-defined outermost layer of hydrophobin-borne rodlet bundles (40). Revealed by scanning electronic microscopy (SEM), such a conidial coat was intact for the control strains, contrasting to impairment or disappearance of most rodlet bundles on conidial surfaces of the Δ*cfp* mutant (Fig. 4F), although conidial size and complexity differed insignificantly between the mutant and control strains (Fig. 4G). In the Δ*cfp* mutant, the impaired conidial coat coincided well with reduced hydrophobicity. Both altered carbon epitopes and impaired rodlet bundles on conidial surfaces suggested an important role of CFP in sustaining cell wall integrity and escaping PAMPs from host receptors to minimize host immune responses.

### Profound effect of CFP on genomic expression.

**FIG 5:**
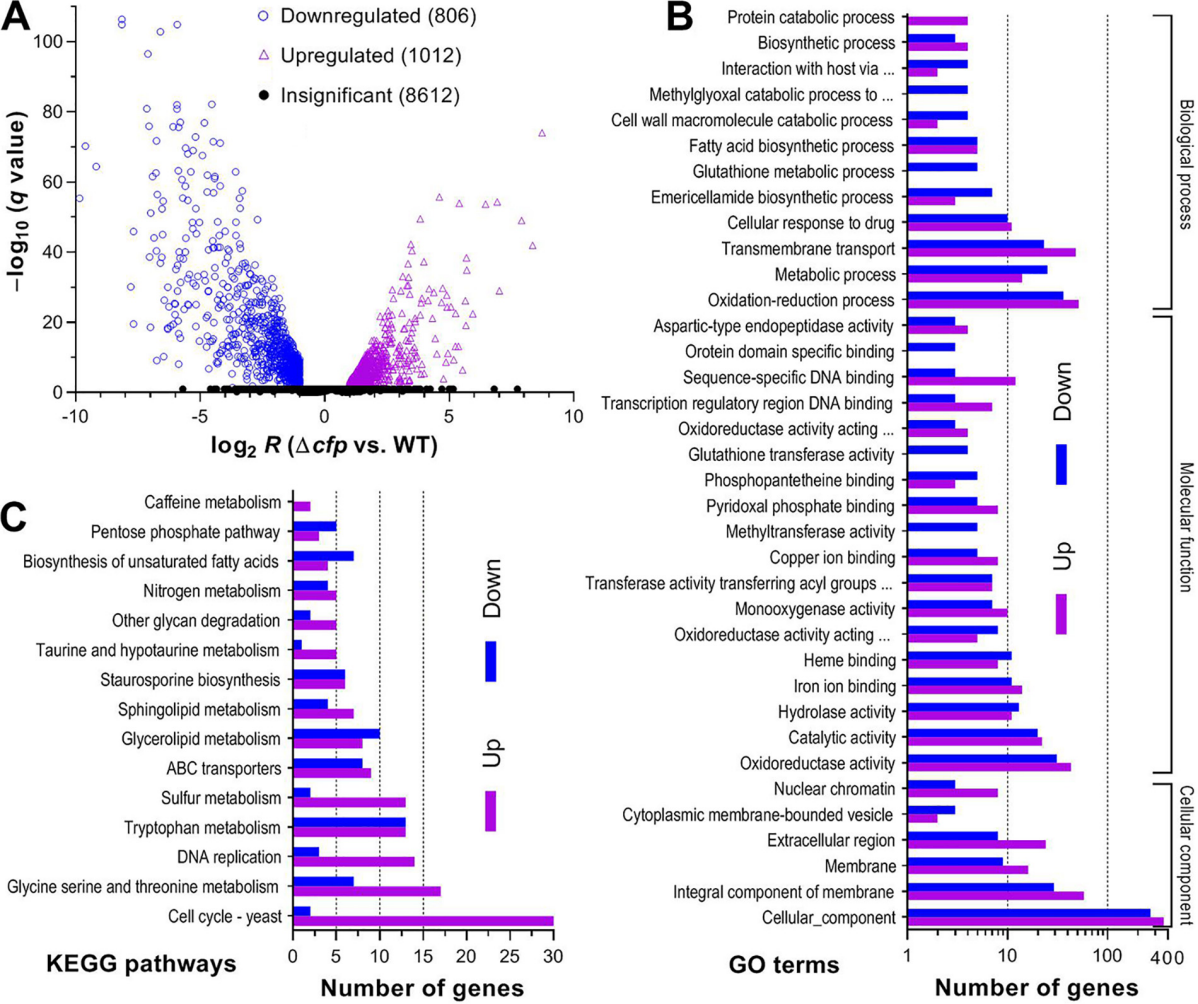
Profound effect of *cfp* disruption on genomic expression of B. bassiana. (A) Distributions of log_2_ *R* and *q* values for all genes identified in the transcriptomes, which were generated from three 3-day-old SDAY cultures (replicates) of Δ*cfp* and WT strains grown at the optimal regime of 25°C and L:D 12 h:12 h. Differentially expressed genes (DEGs) are those significantly downregulated (log_2_ *R* ≤ −1) or upregulated (log_2_ *R *≥ 1) at the level of *q *value of <0.05. The rest of the genes are insignificantly affected (−1 ≤ log_2_ *R *≤ 1 or *q *≥ 0.05 when log_2_ *R* ≤ −1 or ≥ 1). (B and C) Counts of DEGs significantly enriched (*P* < 0.05) for three function classes (main GO terms shown) and KEGG pathways.

For in-depth insight into the effects of CFP on pathogenicity and virulence, transcriptomes were constructed based on three 3-day-old cultures (replicates) of the Δ*cfp* and WT strains grown on cellophane-overlaid SDAY plates at the optimal regime. The resultant data set comprised 10,430 genes mapped to the B. bassiana genome (5) or not mapped (Fig. 5A). Identified from the data set were 1,818 differentially expressed genes (DEGs; up/down ratio, 1,006:801) at significant levels (log_2_ *R* ≤ –1.00 or ≥ 1.00, *q *< 0.05), including six and five expressed only in Δ*cfp* and WT strains, respectively (see Table S1). These DEGs comprised 17.54% of the whole genome. Notably, 423 DEGs (up/down ratio, 204:219) encode hypothetical proteins, and another 293 (up/down ratio, 120:173) are not annotatable, including 127 new genes likely involved in the CFP-mediated cellular events.

10.1128/mSystems.00098-21.4 TABLE S1 List of DEGs in the Δ*cfp* versus WT strains of B. bassiana. Download Table S1, XLSX file, 0.9 MB. Copyright © 2021 Mou et al. 2021 Mou et al. https://creativecommons.org/licenses/by/4.0/ This content is distributed under the terms of the Creative Commons Attribution 4.0 International license.

Gene ontology (GO) analysis resulted in significant enrichments of 1,665 DEGs (up/down ratio, 1,003:662) to 15, 23, and 63 GO terms of three function classes (see Table S2). Shown with main GO terms in Fig. 5B, the cellular component class comprised 818 DEGs (up/down ratio, 495:323), which were mostly enriched to the terms cellular component, integral component of membrane, membrane, extracellular region, nuclear chromatin, and membrane-bounded vesicle and eisosome. Enriched to molecular function class were 428 DEGs (up/down ratio, 252:166) involved mainly in oxidoreductase activity, catalytic activity, iron/copper ion binding, hydrolase activity, heme binding, monooxygenase activity, sequence-specific DNA binding, transferase activity, pyridoxal phosphate binding, transcription regulatory region DNA binding, and phosphopantetheine binding. Enriched to biological process were 429 DEGs (up/down ratio, 256:173) involved mainly in the processes of oxidation-reduction, transmembrane transport, metabolism, cellular response to drug, emericellamide biosynthesis, fatty acid biosynthesis, biosynthesis, protein catabolism, glutathione metabolism, interaction with host via protein secreted by type II secretion system, DNA replication initiation, cell wall macromolecule catabolism, and amino acid metabolism. This class also included the smaller, but important, terms (3 to 5 DEGs each) methylglyoxal catabolism, phospholipid biosynthesis, mitotic spindle assembly, regulation of nitrogen utilization, vacuolar transport, polyphosphate metabolism, Zn^2+^ transmembrane transport, lysosomal microautophagy, cellular protein modification, RNA catabolism, and biosyntheses of several secondary metabolites.

10.1128/mSystems.00098-21.5 TABLE S2 GO analysis of DEGs in the Δ*cfp* versus WT strains of B. bassiana. Download Table S2, XLSX file, 0.1 MB. Copyright © 2021 Mou et al. 2021 Mou et al. https://creativecommons.org/licenses/by/4.0/ This content is distributed under the terms of the Creative Commons Attribution 4.0 International license.

Kyoto Encyclopedia of Genes and Genomes (KEGG) analysis resulted in 215 DEGs (up/down ratio, 141:74) enriched to 15 pathways (Fig. 5C; Table S3). Counts of enriched DEGs were 32 in cell cycle, 26 in tryptophan metabolism, 24 in glycine, serine, and threonine metabolism, 18 in glycerolipid metabolism, 17 in DNA replication, 17 in ABC transporters, 12 in sulfur metabolism, 11 in sphingolipid metabolism, 11 in biosynthesis of unsaturated fatty acids, 9 in nitrogen metabolism, 8 in pentose phosphate pathway, 7 in other glycan degradation, 6 in taurine and hypotaurine metabolism, and 2 in caffeine metabolism.

10.1128/mSystems.00098-21.6 TABLE S3 KEGG enrichment analysis of DEGs in the Δ*cfp* versus WT strains of B. bassiana. Download Table S3, XLSX file, 0.03 MB. Copyright © 2021 Mou et al. 2021 Mou et al. https://creativecommons.org/licenses/by/4.0/ This content is distributed under the terms of the Creative Commons Attribution 4.0 International license.

So many genes involved in a wide array of functions and pathways were dysregulated in the absence of *cfp*, indicating a profound effect of CFP on the genomic expression in B. bassiana.

### Transcriptomic insights into pleiotropic effects of CFP.

Both GO and KEGG enrichment analyses revealed several dozens of DEGs involved in the oxidoreductase activity and oxidation-reduction process, coinciding well with reduced SOD and catalase activities and increased sensitivities to menadione and H_2_O_2_. Thirty-two DEGs were involved in the cell cycle pathway and likely associated with impeded proliferation of hyphal bodies *in vivo* and reduced blastospore production *in vitro*, revealing an effect of the disturbed cell cycle on the mutant virulence attenuated via CBI. Another 32 DEGs (up/down ratio, 17:15) were involved in cuticle degradation and fungal pathogenicity via NCI (see Table S4). These DEGs encode cutinases, chitinases, subtilase-like proteins, subtilisin-like proteases, glycoside hydrolases, and secreted lipases aside from bassianolide nonribosomal peptide synthetase and beauvericin biosynthetic protein likely involved in insecticidal activity (41–43).

10.1128/mSystems.00098-21.7 TABLE S4 Lists of DEGs associated with phenotypes and cellular events in the Δ*cfp* mutant. Download Table S4, XLSX file, 0.1 MB. Copyright © 2021 Mou et al. 2021 Mou et al. https://creativecommons.org/licenses/by/4.0/ This content is distributed under the terms of the Creative Commons Attribution 4.0 International license.

Also listed in Table S4, 29 DEGs (up/down ratio, 13:16) encode proteins involved in cell wall integrity, while eight others (up/down ratio, 2:6) encode heat shock proteins (HSPs) involved in responses to heat shock and other stresses (17, 44–46). Unexpectedly, the hydrophobin genes *hyd1* (BBA_03015) and *hyd2* (BBA_00530) required for biosynthesis and assembly of hydrophobin rodlet bundles (40) were upregulated. Moreover, 148 DEGs (up/down ratio, 114:34) encode varieties of transporters, permeases, ATPases, and analogs, which are all vital for maintenance of vacuolar/cellular homeostasis and cellular transport of nutrients, metal cations, and xenobiotics and also for multiple stress responses outside and inside host insect, suggesting a role of CFP in the cellular processes. Eleven DEGs were involved in autophagy, mitophagy, phagocytosis, and endocytosis, although none of them encodes an ATG protein known in fungal insect pathogens (47). Thirty-two DEGs (up/down ratio, 23:9) encode proteins in various signaling pathways, including 10 involved in the mitogen-activated protein kinase (MAPK) pathways, although none of them is a component of the Hog1, Slt2, and Fus3 cascades that mediate asexual development, multiple stress responses, and/or virulence (23, 39). Similarly, none of 11 DEGs (up/down ratio, 7:4) presumably involved in fungal development and sporulation is the CDP gene *brlA* or *abaA* required for conidiation and dimorphic transition (19), hinting at some other factors responsible for the Δ*cfp* defects in aerial conidiation and submerged blastospore production. Additionally, many more DEGs (up/down ratio, 146:121) were involved in biogenesis, biosynthesis, and carbon/nitrogen metabolisms, implicating pleiotropic effects of CFP on the fungal life cycle.

Notably, 58 DEGs (up/down ratio, 34:24) encode transcription factors and analogs likely involved in direct transcription mediation. Many more DEGs (up/down ratio, 126:49) also encode proteins/enzymes as direct and/or indirect gene regulators, since they function in posttranslational modifications, RNA structure and function, and DNA replication, repair, binding, and regulatory activity as well as in translation. The dysregulation of so many genes involved in transcriptional mediation highlights a genome-wide effect of CFP on the fungal life cycle.

### CFP has no DNA-binding activity.

To elucidate the genome-wide effect, electrophoretic mobility shift assays (EMSAs) were performed to probe whether purified CFP extract could bind promoter DNA fragments of 12 genes sharply repressed in the Δ*cfp* mutant (see Fig. S3A). Agarose gel electrophoresis showed no shifting sign for any DNA fragment bound by uploaded CFP samples (0.8 to 4.0 μg) and also no shifting sign for any CFP sample bound to the DNA sample in the gels stained with Coomassie brilliant blue (Fig. S3B). The nonshifting signals of gradient CFP samples in binding the tested DNA samples were distinguished from shifting signals of the promoter DNA extract of *ole1* (Δ9-fatty acid desaturase gene) bound by gradient HapX (bZIP transcription factor) samples as a positive control (Fig. S3C) but similar to nonshifting signals of the *ole1* DNA reacting with gradient thioredoxin (Trx1) samples as a negative control (Fig. S3D) (48). These data indicate an inability for CFP to bind the promoter regions of potential target genes for their transcription mediation.

10.1128/mSystems.00098-21.3 FIG S3 Electrophoretic mobility shift assay (EMSA) for DNA-binding activity of CFP in B. bassiana. (A) Paired primers used for amplifying from the WT DNA the promoter DNA fragments of 12 genes, which were drastically repressed in the Δ*cfp* mutant. (B) EMSA for the binding activity of CFP to each of 12 promoter DNA fragments amplified. CFP was extracted from the cell lysate of Escherichia coli expressing *cfp* and purified through affinity chromatography column, dialysis, and concentration. Aliquots of 4 μl DNA extract (100 ng/ml) were loaded for a 30-min reaction with 0.8, 1.6, 2.4, 3.2, and 4.0 μg (lanes 2 to 6, respectively) of CFP extract through agarose gel electrophoresis (top black/white panels). Lanes 1 and 7 were loaded with only 4 μl DNA extract and only 4 μg CFP extract, respectively, and used as negative controls. (C and D) EMSAs for the respective binding activities of purified HapX (transcription factor as positive control) and Trx1 (negative control) to the promoter DNA of *ole1*. Lanes 2 to 7 were loaded with 0.5, 2, 3.5, 5, 7.5 and 9 μg of each protein, respectively, for 30-min reaction with 4-μl aliquots of *ole1* DNA extract (lane 1, 4 μl DNA alone; lane 8, 9 μg protein alone). All gels were stained with coomassie brilliant blue (bottom blue panels) to show whether purified CFP was bound to the promoter DNA fragments tested. Note no signal for the DNA-binding activity of any uploaded CFP sample. Download FIG S3, JPG file, 1.0 MB. Copyright © 2021 Mou et al. 2021 Mou et al. https://creativecommons.org/licenses/by/4.0/ This content is distributed under the terms of the Creative Commons Attribution 4.0 International license.

## DISCUSSION

CFP homologs seem to exist only in certain asexual entomopathogens of Hypocreales. In B. bassiana, unique CFP in a list of SSPs was postulated to function during host infection (32) and proved essential for insect pathogenicity via NCI and virulence via CBI in this study. Both NCI and CBI bioassay data highlight that CFP is not only a super virulence factor but also a core regulator of the insect-pathogenic life cycle. As NSP-bearing SSPs, cysteine-rich VLP4 and cysteine-free CypB were previously characterized as virulence factors due to their marked contributions to the fungal kill action via NCI (33, 34). However, nine other cysteine-rich or -deficient SSPs were dispensable for the fungal virulence in this study. The previous and present studies indicate that SSPs presumably involved in fungus-insect interactions do comprise, but are not necessarily, virulence effectors. As a cysteine-free SSP, CFP is very different from VLP4 and those elucidated as virulence factors in plant-pathogenic fungi (28–31). The fact that CFP::GFP was localized in the nucleus but extracellularly undetectable indicates that NSP-bearing CFP is not a secreted protein. Its pleiotropic effect on B. bassiana is discussed below.

The fungal insect pathogenicity via NCI can be abolished or compromised severely in different fashions. First, impeded hyphal growth on scant media resembling oligotrophic insect surfaces may inhibit or slow hyphal invasion into the host body. As examples, NCI was abolished in the absence of *fus3*, *ste7*, or *ste11* in the MAPK Fus3 cascade (23, 49) and of *ssr4* encoding a nuclear cosubunit of chromatin-remodeling SWI/SNF and RSC complexes (37) and greatly compromised in the absence of *rei1* encoding a cytoplasmic pre-60S subunit export factor (36). A similar situation occurred when histone H3 was hypo- or hyperacetylated by loss-of-function mutation of histone acetyltransferase Gcn5 or deacetylase Rpd3 (20, 21). The mutants of these genes showed severe growth defects on scant media and reduced conidiation capacity due to sharp repression of CDP genes or dysregulation of many genes involved in carbon/nitrogen metabolism. Second, an incapability of hyphal invasion into the host body is related to no conceivable defect in hyphal growth but tied to abolished aerial conidiation and submerged blastospore production due to inactivation of CDP by *brlA* or *abaA* disruption (19). In our Δ*cfp* mutant, abolished NCI was attributable mainly to blocked secretion of cuticle-degrading enzymes (10) rather than to either an accountable growth defect or repressed expression of CDP or Fus3-cascaded genes. Our transcriptomic analysis revealed 32 dysregulated genes encoding NCI-required enzymes/proteins. Taken together with the incapable growth of the mutant out of insect cadavers, we infer that CFP may serve as an active player in synthesis and secretion of diverse cuticle-degrading enzymes and hence is indispensable for insect pathogenicity of B. bassiana via NCI.

The Δ*cfp* virulence attenuated via CBI indicates a pivotal role of CFP in postinfection cellular events crucial for virulence. The transcript-declining trend of *cfp* in the submerged TPB cultures during the 4-day incubation suggests its role in the fungal proliferation *in vivo*. Upon entry into host hemocoel, dimorphic transition is critical for intrahemocoel proliferation by yeast-like budding to accelerate insect death from mycosis development. In the Δ*cfp* mutant, blocked dimorphic transition was revealed by both the absence of hyphal bodies *in vivo* and a 61% decrease of blastospore production in the TPB cultures. Moreover, the mutant conidia were coated with increased amounts of carbohydrate epitopes and impaired hydrophobin rodlet bundles (41). Our transcriptomic data comprised dozens of dysregulated genes involved in cell wall integrity. Of those, upregulation of *hyd1* and *hyd2* suggest other cell wall-related genes that could be associated with an impaired coat and reduced hydrophobicity of the mutant conidia, such as those genes involved in biogenesis and biosynthesis, although their roles in hydrophobin synthesis and assembly remain unclear. For the Δ*cfp* mutant, both altered carbohydrate epitopes and dysregulated genes implicate that impaired surfaces of injected conidia could have more PAMPs exposed to host receptors (9), which could lead to more intensive host immunity responses to be overcome by longer interaction *in vivo*. This implication hints at a possible role of CFP in hiding PAMPs to minimize host immune responses during NCI and hemocoel colonization. Additionally, bassianolide nonribosomal peptide synthetase and beauvericin biosynthetic protein are known as the fungal virulence factors (41–43). The drastic repression of their coding genes suggests reduced secretion of such insecticidal metabolites by the Δ*cfp* mutant, thereby helping to explain a delay of its lethal action against the injected larvae.

In the Δ*cfp* mutant, compromised conidiation and blastospore production were not due to repressed CDP genes. Instead, the mutant had several hundreds of dysregulated genes involved in carbon/nitrogen metabolism, transmembrane transport, cellular homeostasis, signal transduction, autophagy, phagocytosis, and endocytosis aside from those involved in cell cycle. Dysregulation of these genes suggests a core role of CFP in diverse cellular processes and events which are also influential on asexual development and stress responses. The core role is likely linked to a nuclear localization of CFP and its predicted activity in association with various organelles. However, nuclear CFP had no DNA-binding activity in EMSAs and hence was not capable of directly regulating gene transcription by itself. Previously, coding genes of Ssr4 and Rei1 were increasingly upregulated during host infection by B. bassiana (32). Like CFP, Ssr4 and Rei1 had no DNA-binding activity but orchestrated transcription of 2,517 and 1,400 genes, which were dysregulated in the respective Δ*ssr4* and Δ*rei1* mutants displaying severe defects in growth, conidiation, pathogenicity, and virulence (36, 37). Many of those genes were involved in carbon/nitrogen metabolisms and transport of nutrients, and dozens were involved in transcription, translation, and posttranslational modifications; but none were involved in CDP. The genome-wide regulatory roles of Ssr4 and Rei1 were speculated to rely upon a role of Ssr4 as a cosubunit of the SWI/SNF and RSC complexes that can bind the binding sites of transcription factors (50, 51) and of Rei1 in orchestrating dissociation and recycling of nucleocytoplasmic pre-60S factors (52–54). In this study, the Δ*cfp* mutant had many more dysregulated genes (233) involved in direct and indirect gene regulation than those seen in the Δ*ssr4* and Δ*rei1* mutants. This offers insight into the dysregulation of another 1,585 genes, nearly half (up/down ratio: 324/392) of which encode hypothetical or unknown proteins. So many of the unknown genes orchestrated by CFP are likely critical for the fungal insect-pathogenic life cycle. However, it is unclear how CFP is tied to dozens of transcription factors required for gene regulation and to many more proteins/enzymes that may mediate gene transcription in direct and indirect fashions. Since CFP::GFP was localized in the nucleus and also associated with predicted organelles, we speculate that, like Ssr4 and Rei1, CFP might act as a component of certain nuclear and/or nucleocytoplasmic protein complexes. This hypothesis opens a door to explore what complexes recruit the component CFP lacking any predictable domain as a clue to clarify its genome-wide regulatory role and why so small of a simple protein has a profound effect on insect pathogenicity and virulence-related cellular events in B. bassiana.

Conclusively, not all SSPs involved in fungus-insect interactions are virulence effectors. Cysteine richness is not a necessary criterion for SSPs to be considered so. This is unveiled by indispensability of CFP and dispensability of nine other cysteine-rich or -deficient SSPs for the fungal virulence and offers novel insight into roles of fungal SSPs in the insect-pathogenic life cycle.

## MATERIALS AND METHODS

### Recognition and bioinformatic analysis of fungal CFP homologs.

The amino acid sequence of CFP found in the previous B. bassiana transcriptome (32) was used as a query to search through the NCBI databases of ascomycetes, including entomopathogenic and nonentomopathogenic fungi, by BLAST analysis (https://blast.ncbi.nlm.nih.gov/Blast.cgi). The located homologs were structurally compared with the query by sequence alignment with DNAman 8.0 at http://www.bio-soft.net/format/DNAMAN.htm/ and conserved domain analysis at https://www.ncbi.nlm.nih.gov/Structure/ or http://smart.embl-heidelberg.de/, followed by phylogenetic analysis with a maximum likelihood method in MEGA7 at http://www.megasoftware.net/. NPS and NLS were predicted from each protein sequence at http://www.cbs.dtu.dk/services/SignalP-4.1/ or http://nls-mapper.iab.keio.ac.jp/. Possible extra- and intracellular activities of B. bassiana CFP were predicted with WoLF PSORT (https://wolfpsort.hgc.jp/).

### Subcellular localization and transcriptional profiling of CFP in *B.* bassiana.

The coding sequence of *cfp* (BBA_02121) was amplified from the WT cDNA with paired primers (see Fig. S2B in the supplemental material) and inserted into linearized pAN52-GFP-bar (33) for fusion of *cfp* to the N terminus of *gfp* (U55763), forming *cfp*::*gfp* in the plasmid vectoring P*gpdA* (promoter) and T*trpC* (terminator). The plasmid was transformed into WT via blastospore transformation, resulting in putative transformants screened by *bar* resistance to phosphinothricin (200 μg/ml). A transgenic strain showing strong green signal was incubated on SDAY (4% glucose, 1% peptone, and 1.5% agar plus 1% yeast extract) at 25°C and L:D 12 h:12 h or 0 h:24 h for conidiation. Collected conidia were suspended in SDBY for a 48-, 58-, or 60-h incubation on a shaking bed (150 rpm) at 25°C. LSCM was applied to determine subcellular localization of CFP::GFP expressed in the hyphae of the 60-h-old culture stained with DAPI (4′,6′-diamidine-2′-phenylindole dihydrochloride; Invitrogen) or in the conidia produced at L:D 12 h:12 h or 0 h:24 h but not stained. Subcellular responses of CFP::GFP to stress cues were examined after 48-h-old hyphae were shaken for 12 h in CDB (3% sucrose, 0.3% NaNO_3_, 0.1% K_2_HPO_4_, 0.05% KCl, 0.05% MgSO_4_, and 0.001% FeSO_4_) lacking the carbon and nitrogen sources or 58-h-old hyphae were triggered for 90 min with 4 mM H_2_O_2_ or 0.4 M NaCl in one-fourth SDBY. Alternatively, 58-h-old hyphae were suspended in 0.8-mm-thick PBS, followed by exposure to UVB irradiation of 1 J/cm^2^ (weighted wavelength, 312 nm). The hyphae from each treatment were stained with DAPI, followed by LSCM analysis. ImageJ software (https://imagej.nih.gov/ij/) was used to measure green fluorescence intensities in the cytoplasm and nuclei of ≥30 cells in 10 hyphae from each treatment. Relative accumulation levels of expressed CFP::GFP in the nuclei of those hyphae were estimated as N/C-GFI ratios. Additionally, supernatants were collected from the 60-h-old cultures of the transgenic and WT strains by filtration and centrifuged for 10 min at 10,000 ×*g*, followed by isolation of nuclear protein extracts from the cells with a nuclear and cytoplasmic protein extraction kit (Beyotime, Shanghai, China). The precipitants and the protein extracts (20 μg uploaded per lane) were probed with mouse monoclonal anti-GFP antibodies (OriGene China, Beijing, China) for the Western blot signal to determine whether CFP::GFP was secreted out of cells or fused as expected.

To examine transcriptional profiles of *cfp* in different WT cultures, 100-μl suspension aliquots (10^7^ conidia/ml) were spread on cellophane-overlaid SDAY plates for a 7-day incubation at the optimal regime or 100-ml aliquots of 10^6^-conidia/ml TPB (CDB amended with 3% trehalose and 0.5% peptone) were incubated for 4 days on the shaking bed. From day 2 onwards, total RNAs were extracted daily from three TPB or SDAY cultures with an RNAiso Plus kit (TaKaRa, Dalian, China) and reversely transcribed into cDNAs with a PrimeScript reverse transcription (RT) reagent kit (TaKaRa). Transcripts of *cfp* in the cDNA samples were assessed via real-time quantitative PCR (qPCR) with paired primers (Fig. S2B) under the action of SYBR Premix *Ex Taq* (TaKaRa). The fungal β-actin gene was used as a reference. A threshold cycle (2^−ΔΔ^*^CT^*) method was used to compute relative transcript levels of *cfp* in the daily WT cultures with respect to the standard on day 2.

### Creation of *cfp* mutants.

The *cfp* mutants were constructed as described previously for targeted *vlp4* disruption and complementation (33). Briefly, the 3′ and 5′ coding/flanking fragments of *cfp* were amplified from the WT DNA and inserted into the restriction enzyme sites of EcoRI/BamHI and XbaI/HpaI in the backbone plasmid p0380-bar, yielding p038-3′cfp-bar-5′cfp for integration into the WT strain for targeted *cfp* disruption through homologous recombination of the *bar*-separated 3′ and 5′ fragments. The full-length coding sequence of *cfp* with flanking regions was amplified from the WT DNA and ligated to the sites of HindIII/XbaI in p0380-sur-gateway to exchange for the gateway fragment, forming p0380-sur-cfp for ectopic integration into an identified Δ*cfp* mutant for targeted *cfp* complementation. Putative mutants were screened by *bar* resistance to phosphinothricin (200 μg/ml) or *sur* resistance to chlorimuron ethyl (10 μg/ml). Expected recombinant events in the colonies were identified via PCR and Southern blot analyses with paired primers and amplified probe (Fig. S2). The positive mutants Δ*cfp* and Δ*cfp*::*cfp* were evaluated together with parental WT in the following experiments of three independent replicates to generate data for one-factor analysis of variance and Tukey’s honestly significant difference (HSD) test for their phenotypic differences.

### Bioassays for fungal virulence.

The virulence of each strain to G. mellonella larvae was assayed through two infection modes. Briefly, three groups (replicates) of ∼35 larvae were immersed for 10 s in 40 ml of a 10^7^-conidia/ml suspension for NCI. For CBI, each larva in each group was injected with 5 μl of a 10^5^-conidia/ml suspension. The control included three groups of larvae immersed or injected with 0.02% Tween 80 used in conidial suspension. All treated groups were kept at 25°C and examined every 12 h for survival records until no more change was observed. LT_50_ was estimated as an index of virulence via either infection mode by probit analysis of the resultant time-mortality trend in each group. Aside from Δ*cfp* and control strains, mutants of nine other SSP genes (shown in Fig. 2B) were constructed using the same strategy as for *cfp* disruption and assayed for their virulence via NCI. These mutants were not studied further due to the lack of virulence change.

### Assessments of cellular events associated with virulence.

A series of cellular events associated with NCI and proliferation *in vivo* were examined to explain abolished pathogenicity and attenuated virulence of the Δ*cfp* mutant in the bioassays. First, larvae that died from injection were incubated at 25°C to observe whether intrahemocoel hyphae can penetrate the host cuticle for outgrowth as an indicator of fungal ability to infect the insect through cuticular penetration. Second, total ECEs and Pr1 activities required for NCI (10) were quantified as units per milliliter from the supernatants of 3-day-old cultures that resulted from shaking incubation of 50 ml 10^6^ conidia/ml CDB-BSA at 25°C and compared with biomass levels as described previously (17, 22). Third, hemolymph samples were taken from surviving larvae 6 days post-NCI and microscopically examined for proliferation status of hyphal bodies. Next, 50-ml aliquots of a 10^6^-conidia/ml TPB mimicking insect hemolymph were incubated for 3 days on the shaking bed at 25°C, followed by measuring blastospore concentration and biomass level to compute dimorphic transition rate (number [no.] of blastospores/mg biomass) in each culture. Flow cytometry was used to assess size and complexity of 2 × 10^4^ blastospores with the readings of forward scatter (FSc) and side scatter (SSc) detectors, followed by fluorescence-activated cell sorter (FACS) analysis to determine G_1_, G_2_, and S phases of the cell cycle in 2 × 10^4^ blastospores stained with the DNA-specific dye propidium iodide.

To explore possible effect of *cfp* disruption on hyphal growth and invasion into a host body, each strain was grown on SDAY, CDA, and CDAs amended with different carbon or nitrogen sources by spotting 1 μl of a 10^6^-conidia/ml suspension per plate for colony initiation. After an 8-day incubation at the optimal regime, the diameter of each colony was estimated as a growth index, with two measurements taken perpendicular to each other across the center. For hyphal responses to stress cues possibly encountered during host infection and hemocoel colonization, aforementioned 8-day colony growth was initiated on CDA containing NaCl (0.7 M) or sorbitol (1 M) for osmotic stress, menadione (0.03 mM) or H_2_O_2_ (2 mM) for oxidative stress, and Congo red (10 μg/ml) or calcofluor white (5 μg/ml) for cell wall-perturbing stress, followed by measuring colony diameters. For response to thermal stress, 2-day-old SDAY colonies initiated at 25°C were exposed to 42°C for 6 h, followed by 6-day growth recovery at 25°C. The sensitivity of each strain to each stress was assessed as percentage of relative growth inhibition ([*D*_c_ – *D*_s_]/*D*_c_ × 100; where *D*_c_ and *D*_s_ are diameters of control and stressed colonies, respectively). Additionally, a catalase activity assay kit (Jiancheng Biotech, Nanjing, China) and SOD activity assay kit (Sigma) were used to assay total catalase and SOD activities (U/mg) in the protein extracts isolated from the 3-day-old SDAY cultures according to the manufacturers’ guides.

To reveal possible effect of *cfp* disruption on conidial quality associated with virulence, 100-μl aliquots of a 10^7^-conidia/ml suspension were sprayed on SDAY plates (9-cm diameter) and incubated for 8 days at the optimal regime. From day 4 onwards, conidial yield was measured as the number of conidia per unit area (cm^2^) of culture from three samples taken from each plate culture with a cork borer (5-mm diameter) as described previously (22). During the period, biomass levels were also measured from cellophane-overlaid SDAY cultures initiated with the same method. Conidial quality was assessed as indices of hydrophobicity in an aqueous-organic system, GT_50_ (h) for 50% germination at 25°C, LT_50_ (min) for tolerance to 45°C wet-heat stress, and LD_50_ (J/cm^2^) for resistance to UVB irradiation (weighted wavelength, 312 nm), as described previously (8, 24, 37). Next, conidia were labeled with the Alexa Fluor 488-labeled lectins ConA, WGA, PNA, and GNL (Vector Laboratories, Burlingame, CA, USA) according to the manufacturer’s guide. Fluorescence intensities of 2 × 10^4^ labeled conidia were assessed as indices of carbohydrate epitopes through FACS analysis. Mean size and complexity of 2 × 10^4^ conidia were also assessed with the FSc and SSc readings from flow cytometry. Finally, the integrity of conidial coat was shown with SEM images.

### Transcriptomic analysis.

Three 3-day-old cultures (replicates) of Δ*cfp* and WT strains grown on cellophane-overlaid SDAY plates were prepared as aforementioned and sent to Lianchuan BioTech Co. (Hangzou, China) for construction and analysis of a CFP-specific transcriptome. Total RNA extraction from each culture, isolation of mRNA from total RNA, fragmentation of mRNA, and syntheses of first- and second-strand cDNAs were as described previously (36). Each double-stranded cDNA was purified and end repaired by adding a single adenine to its end. The final cDNA library was sequenced on an Illumina NovaSeq 6000 platform.

Clean tags generated by filtration of all raw reads from the sequencing were mapped to the B. bassiana genome (5). All data were normalized as fragments per kilobase of exon per million fragments mapped (FPKM). All DEGs were identified at significant levels of both log_2_ *R* of ≤−1 (downregulated) or ≥1 (upregulated) and *q* value of <0.05, annotated with known or putative gene information in the NCBI protein databases, and subjected to GO analysis (http://www.geneontology.org/) for enrichments of GO terms to three function classes (*P* < 0.05) and KEGG analysis (http://www.genome.jp/kegg/) for pathway enrichment (*P* < 0.05).

### EMSAs for binding activity of CFP to promoter DNA fragments.

Due to the profound effect of CFP on genomic expression, EMSAs for protein-DNA interactions (55) were performed to explore whether CFP can bind promoter DNA fragments as described previously (36, 37). Briefly, CFP detected in the cell lysates of Escherichia coli by SDS-PAGE analysis was purified via affinity chromatography column and dialysis and standardized to 0.4 mg/ml. The purified protein extract was used as a template to detect its binding activity to the promoter DNA fragments (amplified from the WT DNA with paired primers in Fig. S3A) of 12 selected genes drastically repressed in the Δ*cfp* mutant. For each DNA extract, 4-μl aliquots (100 ng/μl) were mixed with 0.8, 1.6, 2.4, 3.2, and 4.0 μg (2 to 10 μl) of the CFP extract in 10-μl aliquots of binding buffer comprising 25 mM HEPES (pH 7.4), 50 mM KCl, 5 mM MgCl_2_, 0.5 mM EDTA, 1 mM dithiothreitol, and 5% glycerol. Each EMSA included two negative controls, namely, a combination of 4 μl DNA extract with no protein extract and of 4.0 μg CFP extract with no DNA extract. All of the mixtures were reacted for 30 min at ambient temperature. CFP-bound DNA fragments in the reaction were detected through agarose gel electrophoresis. The gels stained with Coomassie brilliant blue were rinsed repeatedly in washing buffer and visualized for signal of the protein bound to DNA. The signals of the gradient CFP samples in binding each DNA extract were compared with those of the *ole1* (BBA_07664) DNA extract bound by gradient protein samples (0.5 to 9 μg) of the bZIP transcription factor HapX (EJP64582) as a positive control rather than by the samples of the thioredoxin Trx1 (EJP68440) as a negative control (48).

### Data availability.

All data generated or analyzed during this study are included in the paper and associated supplemental files. All RNA sequencing (RNA-seq) data from this study are available at the NCBI’s Gene Expression Omnibus under the accession no. GSE158758.
