# Supplementary figures and images for: A Small Cysteine-Free Protein Acts as a Novel Regulator of Fungal Insect-Pathogenic Lifecycle and Genomic Expression
Source: mSystems. 2021 Mar 23;6(2):e00098-21. doi: 10.1128/mSystems.00098-21 (PMC8546967; doi:10.1128/mSystems.00098-21)

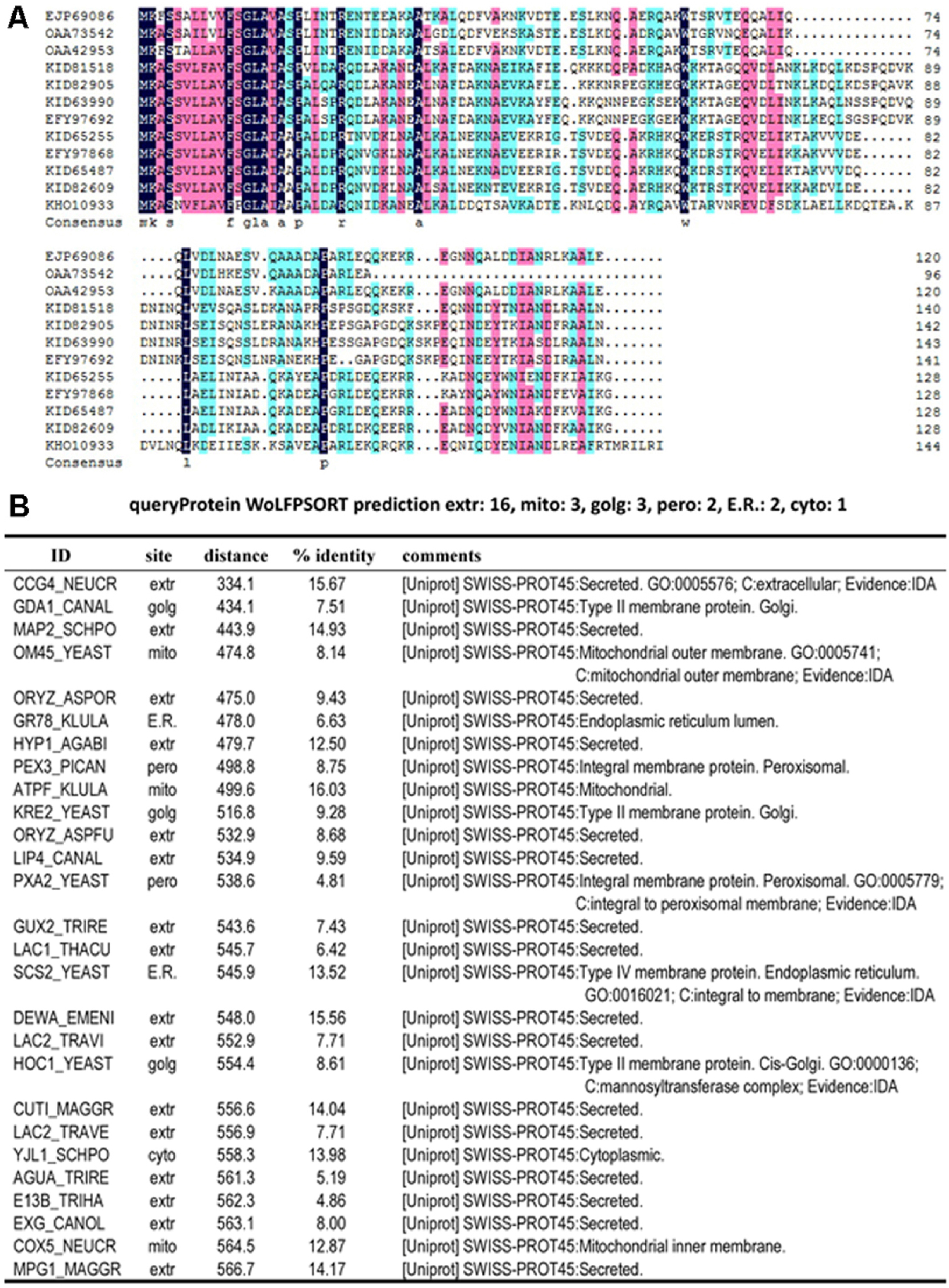

Supplement: FIG S1 [file msystems.00098-21-sf001.jpg]

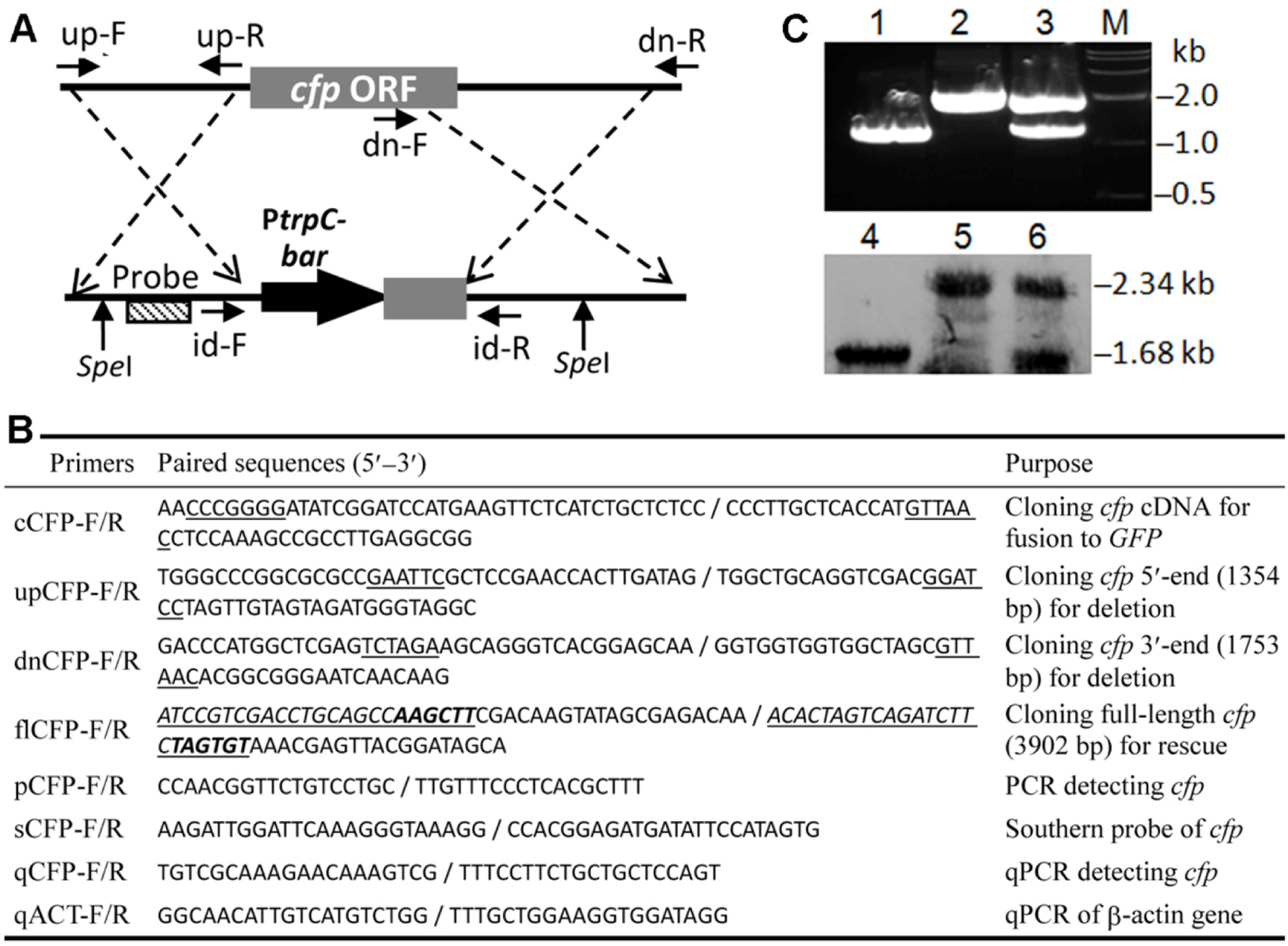

Supplement: FIG S2 [file msystems.00098-21-sf002.jpg]

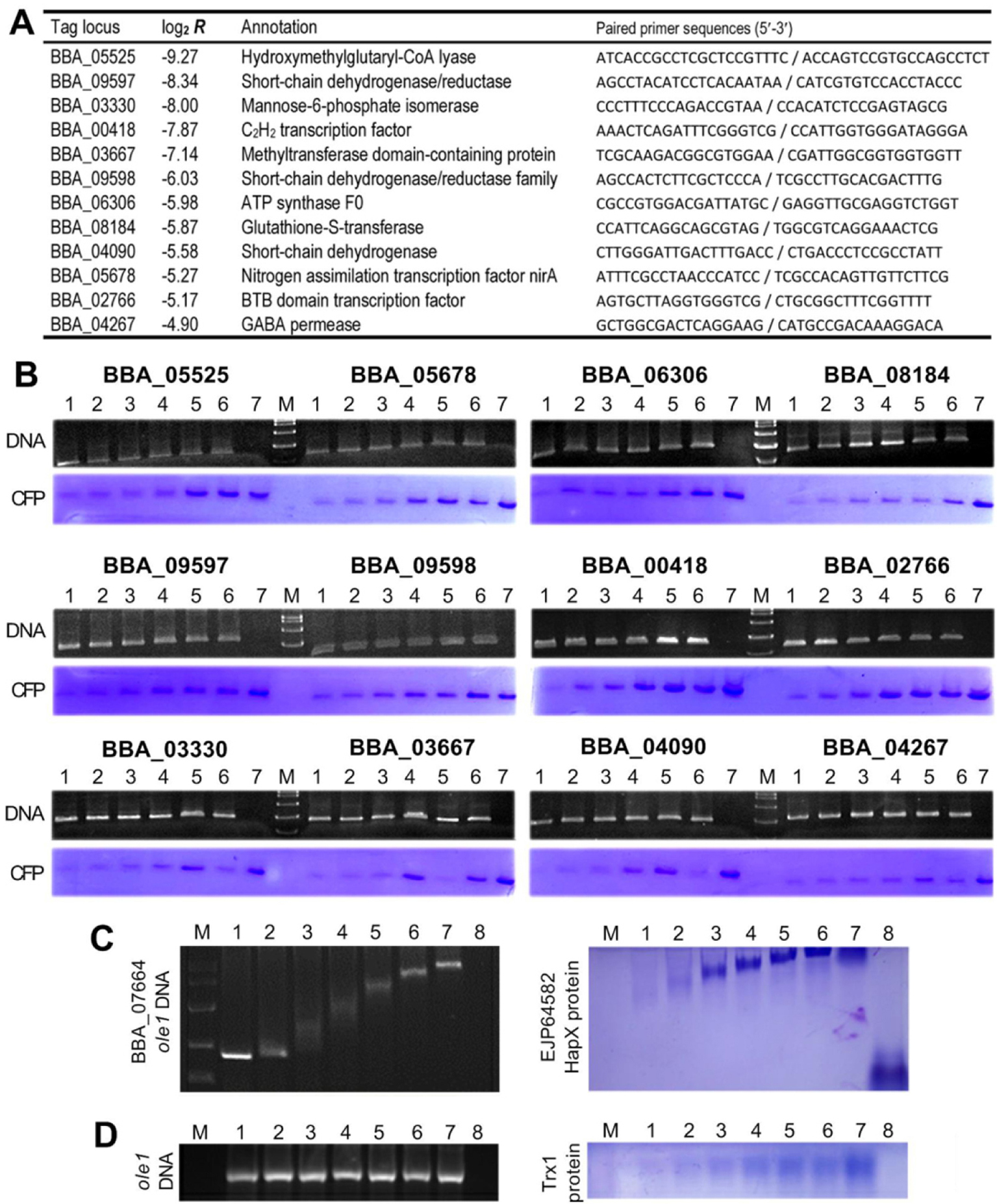

Supplement: FIG S3 [file msystems.00098-21-sf003.jpg]
